# A Parameterized Cross‐Sectional Model for Simulating Balloon Angioplasty in Atherosclerotic Arteries

**DOI:** 10.1002/cnm.70058

**Published:** 2025-07-18

**Authors:** Sanne M. B. Kwakman, Michele Terzano, Malte Rolf, Gerhard A. Holzapfel

**Affiliations:** ^1^ Institute of Biomechanics, Graz University of Technology Graz Austria; ^2^ Department of Structural Engineering Norwegian University of Science and Technology (NTNU) Trondheim Norway

**Keywords:** atherosclerosis, balloon angioplasty, biomechanics, constitutive modeling, parametric model

## Abstract

Atherosclerotic arteries exhibit geometric alterations due to plaque deposition, which often leads to luminal narrowing. Balloon angioplasty is a common and suggested treatment to restore blood flow. However, depending on balloon oversizing, rupture at the plaque shoulder or the fibrous cap may occur. The rupture risk is influenced by factors such as the geometry of the fibrous cap, the lipid pool size, and calcifications. Despite advances in clinical imaging, predicting plaque rupture remains challenging because of lesion variability. This study addresses this gap by identifying key geometrical factors that influence stress distribution during balloon angioplasty, thus improving biomechanical insights and risk assessment. In this work, we develop a parameterized cross‐sectional model of the atherosclerotic artery to investigate the influence of these components on stress distribution during balloon angioplasty. This model can be adapted to different stages and geometries of atherosclerosis. The parametric model enables the evaluation of the influence of uncertain input parameters, especially geometrical parameters, on the outcome of a finite element analysis. Experimental data from a layer‐specific mechanical test on an iliac artery and pressure–diameter curves from balloon inflation tests are used to calibrate the respective constitutive models. Balloon angioplasty is then simulated by inflating a balloon in the narrowed artery without explicitly considering balloon unfolding. We perform 3000 simulations for a local sensitivity analysis by varying the six most influential geometrical parameters and leaving the remaining parameters and the material parameters unchanged. The results show that the amount of the lipid pool has the largest influence on the maximum principal stress in the arterial tissue. Furthermore, the thickness of the fibrous cap plays a critical role in determining the specific location where this maximum occurs. These findings offer valuable insights into potential initiation sites of damage in atherosclerotic arteries.

## Graphical abstract

The parameterized cross‐sectional model allows assessment of the effect of geometric variability in iliac atherosclerotic arteries during angioplasty balloon inflation. The size of the lipid pool and the thickness of the fibrous cap primarily control, respectively, the magnitude and location of the maximum principal stress in the artery. Our study highlights the importance of using a parameterized finite element model over deterministic approaches.

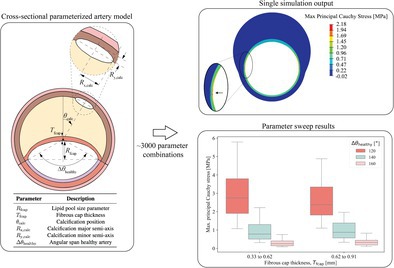

## 1 Introduction

The main cause of cardiovascular disease is atherosclerosis [1], with approximately 70% of acute cardiovascular events caused by rupture of atherosclerotic plaque [2]. In fact, atherosclerosis can also be associated with a number of other cardiovascular diseases, triggering or accelerating their progression. In general, atherosclerosis is a multi‐focal, smoldering immune inflammatory disease affecting the arterial walls. Minimally invasive interventions have become indispensable for the treatment of arterial occlusions due to atherosclerosis. One of the first treatments for lumen obstruction was described by Dotter and Judkins [3] and paved the way for the development of balloon angioplasty and stent placement. According to the 2024 European Society of Cardiology (ESC) Guidelines [4], balloon angioplasty with or without stenting should be considered in patients with symptomatic peripheral arterial disease (Class IIa recommendation). The risk of restenosis, in which the artery narrows again due to plaque formation or scar tissue, has driven the development of drug‐coated and drug‐eluting balloons [5, 6]. This significantly reduces the risk of restenosis, leads to better long‐term outcomes, and reduces the need for repeat interventions.

The adult healthy arterial wall is composed of three distinct layers (intima, media, and adventitia), featuring a complex 3D network of smooth muscle cells, elastin, and bundles of collagen fibrils, see for example [7]. In arteries affected by atherosclerosis, leukocytes and smooth muscle cells invade the intima. The leukocytes remove atherogenic lipoproteins from the intima, leading to the formation of a lipid‐rich core. A fibrous cap, primarily composed of collagen fibers, separates the core from the vessel lumen and influences the vulnerability of the disease. This structure, together with the core, forms an atherosclerotic plaque [8, 9]. As the disease progresses, calcifications become a component of atherosclerotic plaques and appear as both microcalcifications and larger calcified regions called macrocalcifications [10]. Microcalcifications are an important factor in plaque vulnerability as they are thought to contribute to rupture [11]. Stary [12, 13] classified atherosclerotic lesions based on pathological studies, considering factors such as rupture, erosion, fibrous cap thinning, and pro‐thrombotic environments. Although plaques can grow gradually and without symptoms, rupture may suddenly release thrombogenic material into the bloodstream, leading to coronary thrombi in 76% of fatal cases [8, 9]. A plaque prone to rupture is referred to as a vulnerable plaque [14, 15]. These plaques are characterized by a thin, often inflamed, calcified collagenous cap with a depleted population of smooth muscle cells [16, 17, 18, 19, 20]. Beneath the cap is a predominantly lipid‐rich and/or necrotic core surrounded by inflammation and prone to intraplaque hemorrhage [21].

Despite significant advances in clinical imaging techniques that can localize features linked to vulnerability [22, 23] such as cap thickness [24], lipid and/or necrotic core size [25], inflammatory cells [26], calcifications [27], and intraplaque hemorrhage [28], the predictive accuracy of these phenotypic markers for future clinical events remains limited [29, 30, 31]. To enhance the understanding of atherosclerotic plaques and the impact of balloon angioplasty, finite element (FE) analysis has become a powerful tool for modeling these processes in atherosclerotic arteries. Numerous computational studies have explored various aspects: FE modeling of balloon angioplasty with overstretch of non‐diseased tissues [32], plaque fissuring and dissection during angioplasty [33], FE analysis of the interactions between high‐compliance balloon catheters and non‐cylindrical vessels [34], the role of microcalcifications in fibrous cap rupture [11], the importance of modeling balloon folding [35], patient‐specific models of atherosclerotic arteries from virtual histology‐intravascular ultrasound [36], and evaluating the influence of morphological features on the vulnerability of lipid‐rich plaques during stenting [37]. However, a critical gap remains in understanding the influence of variations in artery geometry on simulation outcomes. Atherosclerotic lesions vary considerably between patients, ranging from fatty streaks to complex plaques with varying degrees of calcification, inflammation, and fibrous cap integrity. Nevertheless, the sensitivity of simulation results to geometrical features has not yet been comprehensively investigated.

To address this gap, in this study we develop a parameterized cross‐sectional model to simulate balloon inflation within an atherosclerotic artery. This model allows the incorporation of generally uncertain geometrical parameters, which is crucial for assessing the impact of their high variability on the simulation outputs [38, 39]. In addition, our model enables the assignment of uncertain, component‐specific material parameters derived from imaging and mechanical experiments. This approach allows us to investigate the sensitivity of specific geometrical and material parameters on the output of FE simulations of balloon inflation in atherosclerotic arteries. Our aim is to identify the most influential factors contributing to stress concentrations and potential triggers of plaque rupture and/or damage. In the current study, our modeling approach is applied to iliac arteries, focusing on three important geometrical features: the location of macrocalcifications, the thickness of the fibrous cap, and the size of the lipid pool. The implementation is adaptable to any artery and provides a versatile tool for future research on the biomechanical behavior of atherosclerotic arteries during angioplasty.

## 2 Computational Model

### 2.1 Geometry

**FIGURE 1:**
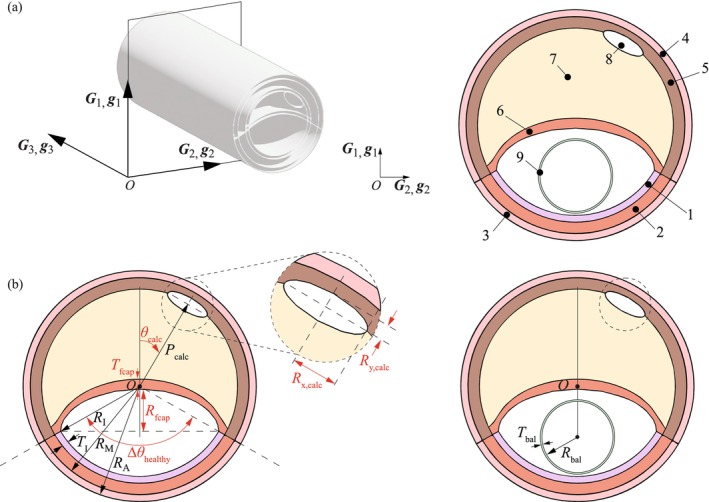
(a) Sketch of the parameterized cross‐sectional plane strain model of the idealized atherosclerotic artery with the various components: (1) healthy intima; (2) healthy media; (3) healthy adventitia; (4) diseased adventitia; (5) fibrotic media; (6) fibrous cap; (7) lipid pool; (8) single macrocalcification; and (9) angioplasty balloon. (b) Representative geometry generated with the Python application programming interface of the Abaqus/Standard FEA software [40], using the main geometric parameters employed in the definition (in red color the parameters that can be varied in the simulations), with luminal intimal radius ($R_{I}$), abluminal medial radius ($R_{M}$), abluminal adventitial radius ($R_{A}$), intimal thickness ($T_{I}$), position of calcification ($P_{\text{calc}}$), semi‐axis of the luminal side of the fibrous cap ($R_{\text{fcap}}$), maximum thickness of the fibrous cap ($T_{\text{fcap}}$), angular position of the calcification ($\theta_{\text{calc}}$), major semi‐axis of the elliptical calcification ($R_{x,\text{calc}}$), minor semi‐axis of the elliptical calcification ($R_{y,\text{calc}}$), and angle spanned by the healthy part of the artery ($\Delta \theta_{\text{healthy}}$). The geometrical parameters in (b) are: $R_{\text{fcap}}=2.8$ mm, $T_{\text{fcap}}=0.9$ mm, $\theta_{\text{calc}}=30{}^{\circ}$, $R_{x,\text{calc}}=1.5$ mm, $R_{y,\text{calc}}=0.5$ mm, and $\Delta \theta_{\text{healthy}}=120{}^{\circ}$.

To gain a more comprehensive understanding of the outcomes associated with balloon angioplasty, various geometrical aspects of atherosclerosis need to be examined. For this purpose, we have developed an idealized model of an atherosclerotic artery, consisting of various entities, as illustrated in Figure 1. In order to optimize the computational time when searching the entire spectrum of randomized parameters, the model is reduced to two dimensions. From a mechanical perspective, we introduce the assumption that the arterial deformation along the axial direction (parallel to the global $G_{3}$ axis in Figure 1a) is neglected, resulting in a plain strain model. Each component of the cross‐sectional model is fully parameterized to allow the study of geometry changes that could correspond to different stages of atherosclerosis; for example, by modifying the size of the lipid pool, the location of calcification, or the thickness of the fibrous cap, as illustrated in Figure 1b. In the figure, parameters that are varied in this work are shown in red. To obtain the geometry shown in Figure 1, the following parameters are required: luminal intimal radius ($R_{I}$), abluminal medial radius ($R_{M}$), abluminal adventitial radius ($R_{A}$), intimal thickness $\left(T_{I}\right)$, position of calcification ($P_{\text{calc}}$), semi‐axis of the luminal side of the fibrous cap ($R_{\text{fcap}}$), maximum thickness of the fibrous cap ($T_{\text{fcap}}$), angular position of the calcification ($\theta_{\text{calc}}$), major semi‐axis of the elliptical calcification ($R_{x,\text{calc}}$), minor semi‐axis of the elliptical calcification ($R_{y,\text{calc}}$) and angle spanned by the healthy part of the artery ($\Delta \theta_{\text{healthy}}$). The arterial model was constructed based on a luminal diameter of 12 mm, which represents to an upper value typical for elderly male patients, who are also the demographic group most commonly affected by iliac atherosclerosis [41].

To simulate balloon angioplasty, a balloon is integrated into the model (see Figure 1b). In the current implementation, the process of unfolding is neglected, so the balloon is described by a circular cross‐section with radius $R_{\mathrm{bal}}$ and thickness $T_{\mathrm{bal}}$, which fits into the lumen of the atherosclerotic artery before the start of the simulation.

### 2.2 Constitutive Behavior

#### 2.2.1 Kinematics in a Co‐Rotational Framework

In the framework of the nonlinear continuum theory of elastic bodies, we introduce the deformation map $\chi \left(X\right)$, which transforms material points $X\in \Omega_{0}$ that are in the stress‐free reference configuration of the continuum body, to spatial points $x=\chi \left(X\right)$ in the deformed configuration Ω [42]. The deformation gradient is defined as the tangent to $\chi \left(X\right)$, with component representation $F_{\mathrm{iJ}}=\partial \chi_{i}/\partial X_{J}=g_{i}\cdot FG_{J}$ with respect to a fixed Cartesian coordinate system $\left\{O,G_{I}\right\}$, with $\left\{g_{i}\right\}\equiv \left\{G_{I}\right\}$.

**FIGURE 2:**
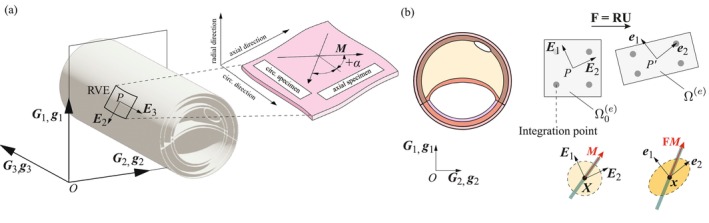
(a) Sketch of the reference configuration of an idealized artery with global basis $\left\{O,G_{I}\right\}$ and local basis $\left\{P,E_{I}\right\}$. The local basis is used to derive the anisotropic mechanical response of the tissue from experiments on a representative volume element (RVE) of the material. (b) The local reference and co‐rotated basis $\left\{P^{\prime },e_{i}\right\}$, adopted in the two‐dimensional FE model. For each element, the local reference basis defined in the element domain $\Omega_{0}^{e}$ at the centroid is transferred to the integration points.

To specify the material symmetry planes, we introduce a local Cartesian coordinate system $\left\{P,E_{I}\right\}$, whose center is a representative volume element of the anisotropic material. As illustrated in Figure 2a, the coordinate axes of this system coincide with the local radial, circumferential, and axial directions of a cylinder. We assume that this local Cartesian basis describes the cylindrical anisotropy of the arterial walls. This is an acceptable approximation provided the size of the rectangular specimens used in the experiments is sufficiently small. To model the anisotropy of a dispersed fiber network, we adopt the concept of generalized structure tensors (GSTs). In the present work, these consist of a symmetric second‐order tensor H, defined by [43].

(1) $$H=\frac{1}{4\pi }\int_{S}\rho \left(N\right)\;N⊗N\;dS$$

where N is the orientation of a single fiber in the reference configuration, with ∥N∥=1. The probability density function (PDF) $\rho =\rho \left(N\right)$ in (1) describes the distribution of collagen fibers over the surface of the unit sphere S. Essentially, the GST weights the contribution of the structure tensor N⊗N of a single fiber by the PDF. The expression of H depends on the class of material symmetry. For example, the anisotropy of a dispersed fiber family with planar mean orientation M is given by the GST as follows: $H=AI+BM⊗M+\left(1-3A-B\right)M_{n}⊗M_{n}$ [44], where A and B are parameters related to the PDF of the fiber orientations. If the mean fiber orientation lies in the axial‐circumferential plane, the unit vectors $M,M_{n}$ are:

(2) $$M=\cos\alpha E_{2}\pm \sin\alpha E_{3},\;M_{n}=E_{1}$$

with the angle α measured counterclockwise with respect to the local circumferential direction $E_{2}$ (see Figure 2a).

For the numerical implementation, it is convenient to introduce a second local basis for the deformed configuration, denoted $\left\{P^{\prime },e_{i}\right\}$ which is derived from a rotation of the basis vectors $E_{I}$. Using the polar decomposition of the deformation gradient F=RU into the symmetric material (right) stretch tensor $U=U^{T}$ and the unimodular rotation tensor R, with $R^{T}=R^{-1}$, we can specify $e_{i}=R_{\mathrm{iI}}E_{I}$ so that $\left\{P^{\prime },e_{i}\right\}$ is known as a *co‐rotated* basis (see Figure 2b). The components of the deformation gradient in the co‐rotational framework are:

(3) $$F_{\mathrm{iJ}}^{R}=e_{i}\cdot FE_{J}=E_{I}\cdot R^{T}FE_{J}=E_{I}\cdot UE_{J}=G_{K}\cdot Q^{T}\mathrm{UQ}G_{L}$$

where $Q_{\mathrm{IJ}}=E_{I}\cdot G_{J}$ is the transformation tensor between the reference global and local bases, and the superscript ${\left(•\right)}^{R}$ denotes the components of tensors in the co‐rotational framework.

Equation (3) shows that the local deformation gradient coincides with the material stretch tensor U [45], which is suitable for formulating constitutive equations of hyperelastic materials. The components of the Cauchy stress tensor σ in the co‐rotational framework are:

(4) $$\sigma_{\mathrm{ij}}^{R}=e_{i}\cdot \sigma e_{j}=e_{i}\cdot \left(2J^{-1}F\frac{\partial \Psi }{\partial C}F^{T}\right)e_{j}=E_{I}\cdot ℱ\left(U\right)E_{J}=G_{K}\cdot Q^{T}ℱ\left(U\right)QG_{L}$$

where $C=U^{2}$ is the right Cauchy‐Green tensor and $ℱ\left(U\right)$ is a constitutive function that depends only on the material stretch tensor U, and the derivatives of the strain‐energy function Ψ [42].

#### 2.2.2 Mechanical Behavior of Atherosclerotic Iliac Arteries

Following Holzapfel et al. [44], the strain‐energy function Ψ of an anisotropic material is given in general terms as:

(5) $$\Psi =\Psi_{\mathrm{vol}}\left(J\right)+\Psi_{\mathrm{iso}}\left({\bar{I}}_{1},{\bar{I}}_{2},{\bar{I}}_{4}^{⋆}\right)$$

where $\Psi_{\mathrm{vol}}$ and $\Psi_{\mathrm{iso}}$ represent the volumetric and isochoric parts of the strain energy, respectively. Here we have tacitly assumed near‐incompressibility and adopted the classical multiplicative split of the deformation gradient into volumetric and deviatoric parts, where the isochoric material stretch tensor is $\bar{U}=J^{-1⁄3}U$, with J=detU. The arguments of the two terms appearing in (5) are the volume ratio J and the isochoric invariants ${\bar{I}}_{1}={\bar{U}}^{2}:I$, ${\bar{I}}_{2}={\bar{U}}^{-2}:I$, and ${\bar{I}}_{4}^{⋆}={\bar{U}}^{2}:H$. Note that the volumetric strain energy $\Psi_{\mathrm{vol}}$ in (5) should only be used as a penalty term to ensure nearly‐incompressible deformations of the anisotropic composite [46]. The isochoric response $\Psi_{\mathrm{iso}}$ is provided by the superposition of a neo‐Hookean term describing the non‐collageneous extracellular matrix of the arterial wall and two families of collagen fibers modeled by an exponential function [44], namely:

(6) $$\Psi_{\mathrm{iso}}=\frac{\mu }{2}\left({\bar{I}}_{1}-3\right)+\frac{k_{1}}{2k_{2}}\sum_{i=1}^{2}\left(\exp\left[k_{2}{\left\langle {\bar{I}}_{4i}^{⋆}-1\right\rangle }^{2}\right]-1\right)$$

The Macaulay brackets $\left\langle •\right\rangle$ in (6) are employed to exclude the mechanical contribution of fibers under compression, so if ${\bar{I}}_{4i}^{⋆}<1$ the anisotropic term in the strain‐energy function is neglected [43].^1^ Equation (6) is governed by the following mechanical parameters: μ, the shear modulus of the ground matrix; $k_{1}$, and $k_{2}$, the stiffness and shape parameters of two mechanically equivalent collagen fiber families. In addition, structural parameters related to the orientation and dispersion of the *i*‐th fiber family are encapsulated in the invariant ${\bar{I}}_{4i}^{⋆}={\bar{U}}^{2}:H_{i}$. Given the strain‐energy functions in (5) and (6), the constitutive function $ℱ\left(U\right)$ appearing in the expression of the co‐rotated Cauchy stress in (4) is:

(7) $$\begin{array}{cc} ℱ\left(U\right)= & \;J^{-1}\mathrm{dev}\left[\mu {\bar{U}}^{2}+2k_{1}\sum_{i=1}^{2}\left(\left\langle {\bar{I}}_{4i}^{⋆}-1\right\rangle \exp\left[k_{2}{\left\langle {\bar{I}}_{4i}^{⋆}-1\right\rangle }^{2}\right]\bar{U}H_{i}\bar{U}\right)\right]\\ & \;+\frac{{\mathrm{d\Psi}}_{\mathrm{vol}}}{dJ\left(U\right)}I \end{array}$$

where $\mathrm{dev}\left(•\right)=\left(•\right)-1/3\mathrm{tr}\left(•\right)I$ is the deviatoric operator.

In the present work, the material and structural parameters are taken from the experimental data documented in Holzapfel et al. [48] for an atherosclerotic iliac artery. To capture the anisotropic behavior of the material, uniaxial tests in the circumferential and axial directions were performed with rectangular tissue strips from individual arterial layers. Specifically, the layers of the healthy artery as well as of the fibrotic media and the fibrous cap were separated. In the absence of specific microscopic investigations, the angle formed by the mean fiber orientation α with the local circumferential direction (see Figure 2a) was fitted to incompressible stretch–stress curves derived from uniaxial tests, assuming no fiber dispersion. Therefore, the GST introduced in (1) is reduced to H=M⊗M. The lipid pool and calcification are described by an isotropic neo‐Hookean model. The parameters used are summarized in Table 1. It should be noted that the two regions labeled 3 and 4 in Figure 1a are modeled as healthy adventitia because the experimental data did not show differences in material behavior between them. However, if future experimental studies reveal variations in material properties, the model can be updated to assign distinct material properties to the different regions. Although material properties naturally influence stress distribution, they were kept constant in this study to isolate the effects of geometric variations. However, incorporating material variability into the model could provide a deeper understanding of the interactions of these factors and their effects on stress distribution.

**TABLE 1:** Summary of the material and structural parameters of the atherosclerotic iliac artery and the angioplasty balloon used in the simulations.

Region	Material parameters			Structural parameter
	*μ* [kPa]	*k*_1_ [kPa]	*k*_2_ [−]	*α* [deg.]
Healthy intima: 1	31.0	51.0	1.10	5.0
Healthy media: 2	15.0	4.0	2.3	7.0
Healthy adventitia: 3, 4	1.75	65.5	61.8	49.0
Fibrotic media: 5	16.2	98.1	10.0	5.0
Fibrous cap: 6	78.9	23.7	26.3	0.0
Lipid pool: 7	0.05			
Calcification: 8	2250			
Balloon: 9	130000			

*Note:* The mean fiber angle α is defined with respect to the local circumferential direction, and the numbers refer to the regions shown in Figure 1a.

#### 2.2.3 Mechanical Behavior of the Angioplasty Balloon

The angioplasty balloon (Crosperio OTW Balloon, Terumo Corporation) is made of Polyamide 11. Experimental pressure–diameter curves from balloon inflation tests are used to identify the mechanical parameters, considering the analytical solution for the inflation of a thin isotropic cylindrical shell [49]. For $\left(R_{o}-R_{i}\right)⁄R_{i}\ll 1$, the inflation pressure can be calculated as:

(8) $$p=\frac{\varepsilon }{{\lambda \lambda }_{z}}\frac{\partial \Psi }{\partial \lambda }$$

where $R_{i}$ and $R_{o}$ are the inner and outer radius of the thin shell, and λ and $\lambda_{z}$ are the radial and axial stretches, respectively.

Based on the available data, the balloon is modeled using a neo‐Hookean model (Table 1). However, it should be noted that the experimental data were limited to a stretch of λ<1.1. During our simulations of balloon inflation (Section 3), the ratio reached approximately λ=2.6, well above the range used in the model calibration.

### 2.3 Numerical Implementation

#### 2.3.1 Finite Element Model

**FIGURE 3:**
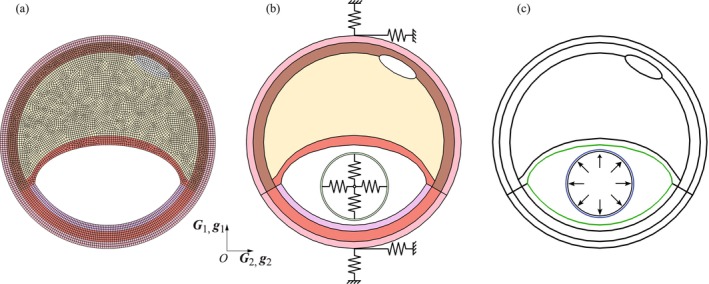
(a) Representative plane strain mesh of the atherosclerotic artery. (b) Boundary conditions for the simulation of balloon inflation. Springs are used to prevent rigid‐body motion of the artery and the balloon. (c) Contact interaction between the inner surface of the atherosclerotic artery and the outer surface of the balloon inflated by inner pressure.

We developed a quasi‐static FE model in the commercial Abaqus/Standard FEA software [40]. Both the atherosclerotic artery and the angioplasty balloon are meshed using plane strain elements (CPE4H, bilinear displacement with constant pressure elements). Based on mesh convergence, a seeding size of 0.13 mm was chosen for the entire geometry (see Figure 3a). The results of the mesh convergence can be found in the Supporting Information.

When simulating balloon angioplasty, certain boundary conditions must be considered to correctly model balloon inflation and its interaction with the vessel wall. As unfolding is neglected, the balloon is placed within the atherosclerotic artery, and rigid‐body motions must be prevented prior to inflation. This is achieved by four springs attached to the balloon, which are connected to a reference point in its center. This reference point is constrained in all directions. Additionally, two springs are connected to the upper and lower ends of the atherosclerotic artery, which are also fixed (see Figure 3b). Inflation is achieved by increasing the radial stress on the inner surface of the balloon. In this study, a maximum value of 2.7 MPa was used. We emphasize that the stated pressure value is not representative of clinical practice but merely the result of the modeling approach and simplifications used to achieve the desired oversizing ratio.

As the balloon expands, it touches the inner walls of the diseased artery. Therefore, contact interactions between the outer surface of the balloon and the inner surface of the artery must be specified (see Figure 3c). For the contact algorithm, the master surface is assigned to the inner surface of the artery (including the luminal side of the healthy intima and the fibrous cap), whereas the slave surface is represented by the outer surface of the balloon. The penalty method available in the Abaqus/Standard FEA software [40] is implemented. Friction was not considered because of missing information.

#### 2.3.2 Local Material Orientation

The adopted anisotropic material law requires a correct definition of the material symmetry planes in the FE model. Following the framework presented in Section 2.2, we use a co‐rotational framework and define the unit vector M of each fiber family with respect to the local reference basis $\left\{P,E_{I}\right\}$, centered at the centroid of each FE. The basis vectors $E_{R}=E_{1}$, $E_{\Theta }=E_{2}$, and $E_{Z}=E_{3}$ identify, respectively, the local radial, circumferential, and axial directions in the reference configuration (see Figure 2a).

To express the unknown local basis vectors $\left\{E_{I}\right\}$ with respect to the global coordinate system $\left\{O,G_{I}\right\}$, in which the approximated element domain $\Omega_{0}^{\left(e\right)}$ is parameterized, a numerical approach is required. In other words, we need to define the transformation tensor $Q_{\mathrm{IJ}}=E_{I}\cdot G_{J}$ for each element of the model (see Figure 2b). We follow a general approach based on the solution of one (in 2D) or two (in 3D) Laplace problems with user‐defined boundary conditions [50]. For the plane strain model, we solve a heat transfer problem in the radial direction. This allows the calculation of the local basis vectors ${\left\{E_{I}\right\}}_{I=1,2}$ in the radial‐circumferential plane from the normalized heat flux vectors at the elements. Further details on the approach and its implementation in the Abaqus/Standard FEA software [40] can be found in the Supporting Information. Note that the definition of the local basis is mesh‐dependent. Therefore, the heat transfer problem must be solved before performing a mechanical analysis and repeated when mesh changes occur.

The output quantities calculated at the integration points, including the strain and stress tensors extracted from the analyses, are provided as matrices expressed with respect to the co‐rotated basis. In the numerical model, this applies to any element for which the local reference basis is defined, regardless of the material symmetry embedded in its constitutive law. However, the choice of the local basis affects the result only for elements where the stress–strain relationship is anisotropic. Finally, it should be recalled that the co‐rotated Cauchy stress tensor can be related to the spatial stress tensor by the transformation $\sigma =\left(\mathrm{QR}\right)\sigma^{R}{\left(\mathrm{QR}\right)}^{T}$, where for the plane strain $\sigma_{i3}^{R}=\sigma_{3i}^{R}=0,i=1,2$.

#### 2.3.3 Parametric Analyses

Inflation of the balloon is simulated with $n_{\mathrm{tot}}=3\;000$ combinations of six geometric parameters, whereas the remaining parameters and the material parameters remain fixed. The parameters that were varied are described below (refer to Figure 1b for illustration):

- $R_{\text{fcap}}$, semi‐axis of the luminal side of the fibrous cap, controlling the size of the lipid pool
- $T_{\text{fcap}}$, maximum thickness of the fibrous cap
- $\theta_{\text{calc}}$, angular position of the calcification
- $R_{x,\text{calc}}$, major semi‐axis of the elliptical calcification
- $R_{y,\text{calc}}$, minor semi‐axis of the elliptical calcification
- $\Delta \theta_{\text{healthy}}$, angle spanned by the healthy part of the artery

**FIGURE A1:**
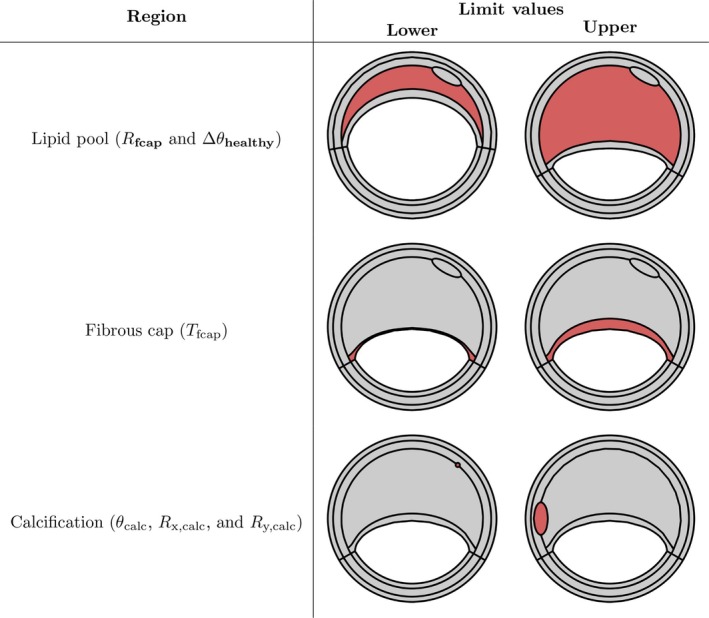
Overview of the possible geometries for the size of the lipid pool, the fibrous cap, and the calcification.

We consolidated the effects of $R_{x,\text{calc}}$ and $R_{y,\text{calc}}$ based on initial simulations by calculating the calcification area as $A_{\text{calc}}=\pi \cdot R_{x,\text{calc}}\cdot R_{y,\text{calc}}$, thus reducing the number of variables. Three values of the parameter $\Delta \theta_{\text{healthy}}$ are considered, corresponding to the angles of 120°, 140°, and 160°. For each case, 1000 simulations are performed by extracting random values from the uniform distributions of the remaining five parameters defined by the ranges given in Table 2. Figure A1 outlines the geometries used, with emphasis on the outlines of the lipid pool, the fibrous cap, and the calcification. The selected parameter ranges ensured that all simulated cases had a stenosis degree between 20% and 60%. These parameters were based on a macroscopic analysis of several human stenotic iliac arteries [8] and provided a realistic representation of the arterial geometry. This allowed us to estimate the plaque severity in different cases and to simulate a range of plausible scenarios for balloon angioplasty. Of the total 3000 simulations, less than 3% failed because of convergence problems and were discarded. The models are generated using a custom script developed for the Python application programming interface (API) of the Abaqus/Standard FEA software and solved using the quasi‐static implicit solver [40]. Each simulation was performed on an Intel(R) Core(TM) i9‐9900K CPU (8 cores), with an average runtime of 2.5 min per simulation. This computational efficiency enabled extensive parametric analyses that would not have been possible with a 3D model.

**TABLE 2:** Parameter ranges for the geometric components of the atherosclerotic artery (see Figure 1b).

Parameter	Range or value	Unit
$R_{\text{fcap}}$	1.8–4.5	mm
$T_{\text{fcap}}$	0.33–1.2	mm
$\theta_{\text{calc}}$	(−90) to (+90)	deg.
$R_{x,\text{calc}}$	0.2–1.5	mm
$R_{y,\text{calc}}$	0.2–0.65	mm
$\Delta \theta_{\text{healthy}}$	120, 140, 160	deg.
$R_{I}$	6.0	mm
$R_{M}$	$1.20R_{I}$	mm
$R_{A}$	$1.28R_{I}$	mm
$T_{I}$	$0.15\left(R_{I}/2\right)$	mm
$P_{\text{calc}}$	$\left(R_{I}+T_{I}\right)\cos\left(\arcsin\left[R_{x,\text{calc}}⁄\left(R_{I}+T_{I}\right)\right]\right)$	mm

The Algorithm 1 summarizes the entire implementation process for generating and analyzing $n_{\mathrm{tot}}$ models of balloon inflation. The process begins with the definition of the input parameters in terms of geometry and material. If $n<n_{\mathrm{tot}}$, the six geometric parameters ($R_{\text{fcap}}$, $T_{\text{fcap}}$, $\theta_{\text{calc}}$, $R_{x,\text{calc}}$, $R_{y,\text{calc}}$, and $\Delta \theta_{\text{healthy}}$) are then defined. Based on the defined parameters, the algorithm creates the geometry of the atherosclerotic artery, the FE mesh, and then determines the local material orientation through a heat transfer analysis. The balloon is then deployed to simulate balloon angioplasty. After assigning the relevant properties and boundary conditions, simulations are carried out. The workflow concludes with the extraction of the simulation outputs, particularly the maximum principal Cauchy stress and its location in the arterial cross‐section. This structured approach ensures a comprehensive and efficient exploration of arterial geometries under different conditions.

ALGORITHM 1 **Framework for Parametric Model Generation and Analysis, With $n_{\mathrm{tot}}$ Samples.**

1: Define input parameters: material (Table 1) and geometry (Table 2)

2: Initialize loop variable n

3: **while** $n<n_{\mathrm{tot}}$ **do**

4: Sample input parameters $R_{\text{fcap}}$, $T_{\text{fcap}}$, $\theta_{\text{calc}}$, $R_{x,\text{calc}}$, $R_{y,\text{calc}}$ from given distributions

5: Generate geometry

6: Generate FE mesh

7: Compute local material orientation

8: Generate balloon geometry

9: Assign material properties and boundary conditions

10: Create input file and submit job

11: **if** job completed **then**

12:  Extract and save output parameters

13: **end if**

14: Update loop variable n+1

15: **end while**

16: **return** Output parameters: principal Cauchy stress $\sigma_{\max}$.

## 3 Results

In this section, we present the findings of FE simulations of balloon inflation in the atherosclerotic iliac artery. The most important result is the absolute maximum and location of the first principal Cauchy stress, as commonly used in other studies [51, 52]. The axis of the first principal stress also corresponds to the circumferential direction of the artery. This information can be directly correlated with the onset of damage to the atherosclerotic arterial wall [53].

**FIGURE 4:**
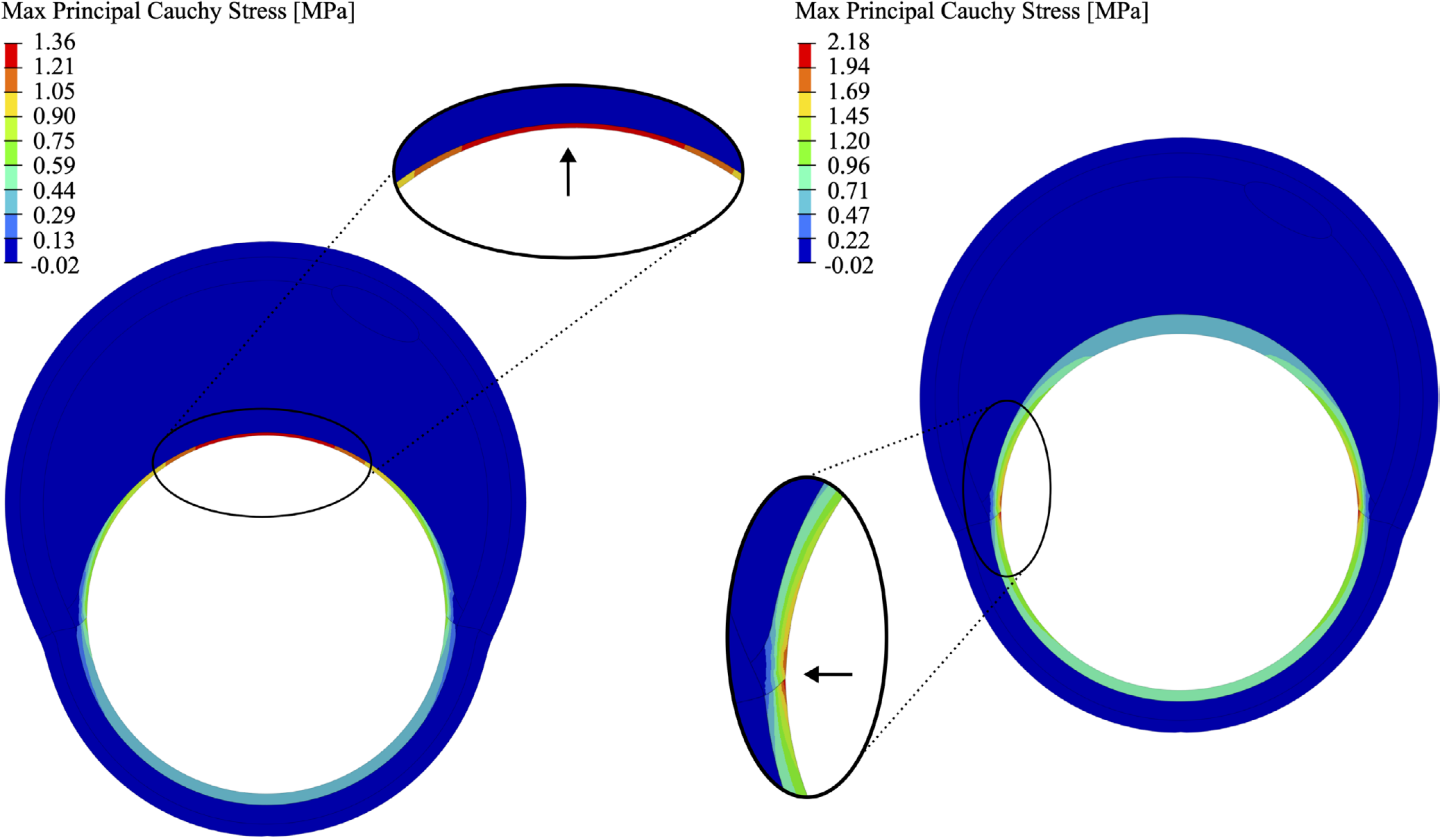
Contours of the maximum principal Cauchy stress from two representative simulations at an inflation pressure of 2.7 MPa (balloon hidden for clarity). The arrows show the location of the highest principal stress: left, at the top of the fibrous cap (T); right, at the shoulder of the atherosclerotic plaque (S). The geometrical parameters of the left geometry are: $R_{\text{fcap}}=4.1$ mm, $T_{\text{fcap}}=0.33$ mm, $\theta_{\text{calc}}=30{}^{\circ}$, $R_{x,\text{calc}}=1.5$ mm, $R_{y,\text{calc}}=0.5$ mm, and $\Delta \theta_{\text{healthy}}=120{}^{\circ}$. For the right geometry the parameters are: $R_{\text{fcap}}=4.1$ mm, $T_{\text{fcap}}=0.9$ mm, $\theta_{\text{calc}}=30{}^{\circ}$, $R_{x,\text{calc}}=1.5$ mm, $R_{y,\text{calc}}=0.5$ mm, and $\Delta \theta_{\text{healthy}}=120{}^{\circ}$. The remaining fixed parameters are reported in Table 2.

Two representative simulation results are shown in Figure 4. Due to variations in the randomly generated geometry, the maximum principal Cauchy stress is concentrated at the top of the fibrous cap (T) in one simulation, while it is predominately localized at the shoulder of the atherosclerotic plaque (S) in the second simulation. In fact, out of the total number of successfully completed simulations, less than ∼2% showed the maximum principal stress located at the top of the fibrous cap. When examining the geometric parameters, we found that the thickness of the fibrous cap in these simulations is at the lower limit of the considered range ($0.33<T_{\text{fcap}}<0.42$ mm). This suggests that in arteries with a thinner fibrous cap, the maximum principal Cauchy stress is more likely to occur at the top, increasing the likelihood of damage initiation in this region.

In the following figures, we plot the results of all simulations in box plots, showing the median, interquartile range, and outliers where present. Recall that each simulation uses the same mechanical parameters, balloon size, and inflation pressure, whereas the geometrical parameters are determined from the distribution of the five parameters considered, as described in Section 2.3. The results are always grouped by lumen size, which is controlled by the angle $\Delta \theta_{\text{healthy}}$. A larger angle indicates a healthier artery, a smaller one indicates a more severe case of atherosclerosis.

**FIGURE 5:**
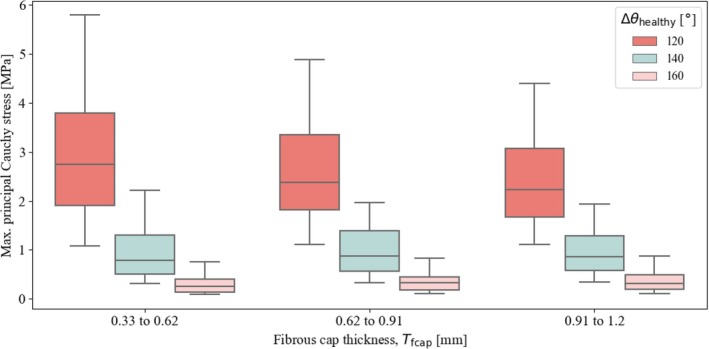
First principal Cauchy stress (absolute maximum) from all simulations, grouped by the thickness of the fibrous cap $T_{\text{fcap}}$, considering three different sizes of the lumen, defined by the angle $\Delta \theta_{\text{healthy}}$.

Figure 5 illustrates the influence of the maximum fibrous cap thickness $T_{\text{fcap}}$, on the simulation results, quantified by the maximum principal Cauchy stress. It is noteworthy that the stress increases as $\Delta \theta_{\text{healthy}}$ decreases. As for the thickness of the fibrous cap, the stress appears to decrease slightly as $T_{\text{fcap}}$ increases. However, for $\Delta \theta_{\text{healthy}}=140{}^{\circ}$, the trend is less pronounced and disappears entirely for $\Delta \theta_{\text{healthy}}=160{}^{\circ}$. Variations in the angle spanned by the healthy part of the artery $\Delta \theta_{\text{healthy}}$ have a significant impact on the stress distribution, with lower values of $\Delta \theta_{\text{healthy}}$ leading to much higher stress levels. It is important to note that the absolute maximum value of approximately 6.0 MPa predicted by the model represents a theoretical limit for the elastic behavior of the tissue. This does not imply an absolute rupture threshold, but rather indicates stress levels at which tissue damage may begin to occur, depending on additional biomechanical and pathological factors. In reality, such stress levels could contribute to tissue weakening and potentially increase the risk of rupture at the site of maximum stress.

**FIGURE 6:**
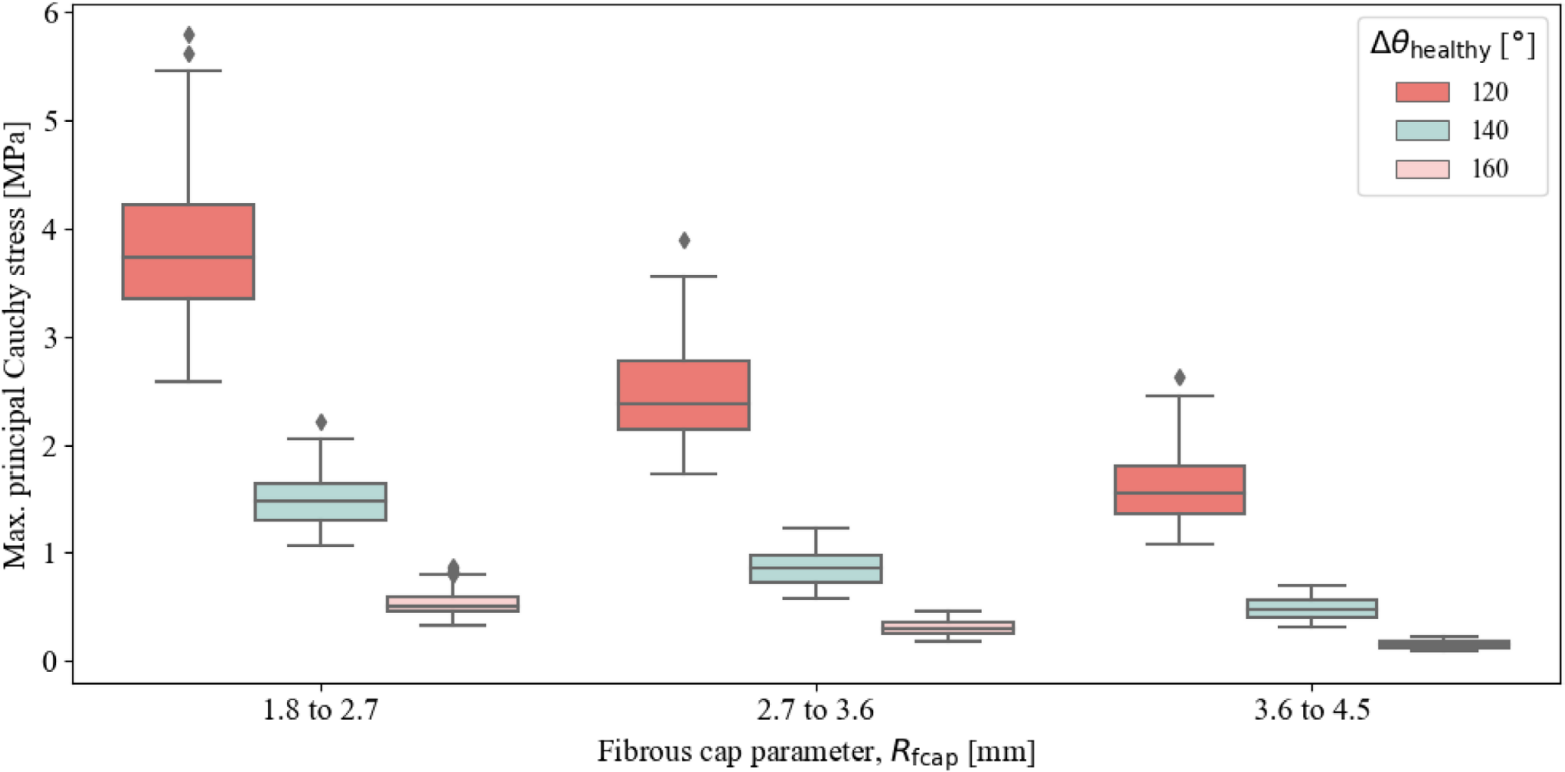
First principal Cauchy stress (absolute maximum) from all simulations, grouped by fibrous cap size $R_{\text{fcap}}$, considering three different sizes of the lumen, defined by the angle $\Delta \theta_{\text{healthy}}$.

Figure 6 demonstrates the influence of lipid pool size on stress. The size of the lipid pool is controlled by adjusting the fibrous cap parameter $R_{\text{fcap}}$ and varying the angle spanned by the healthy part of the artery, $\Delta \theta_{\text{healthy}}$. For the same angle, smaller values of the fibrous cap parameter correspond to larger lipid pools. Focusing on $\Delta \theta_{\text{healthy}}=120{}^{\circ}$, the maximum principal Cauchy stress decreases with increasing $R_{\text{fcap}}$, reflecting the reduction in lipid pool size. These results suggest a linear correlation between the size of the lipid pool, represented by $R_{\text{fcap}}$, and the maximum principal stress. A similar result is observed for the remaining angles. However, as in the previous case, the trend is less pronounced.

**FIGURE 7:**
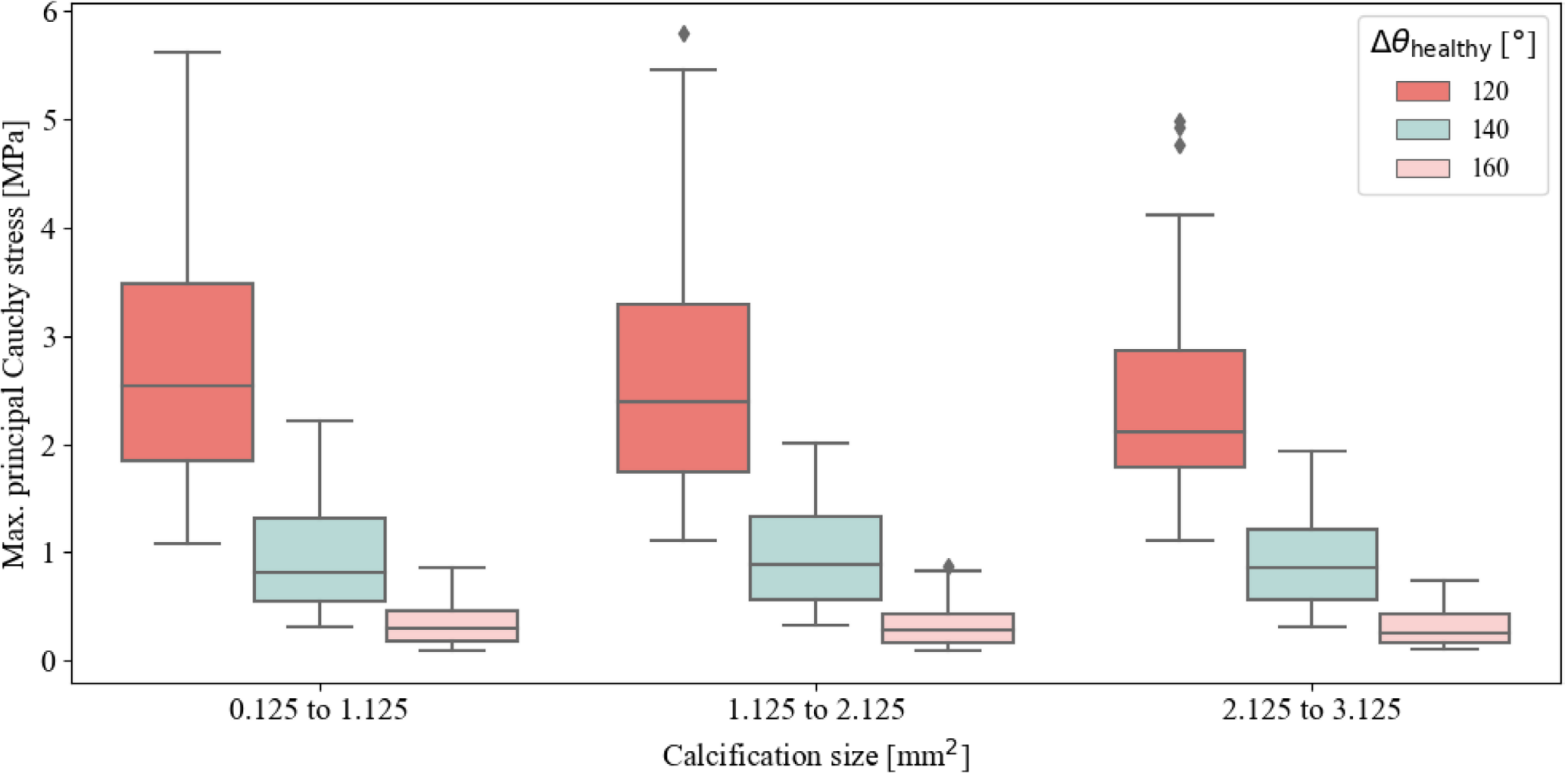
First principal Cauchy stress (absolute maximum) from all simulations, grouped by calcification size quantified by the elliptical area $A_{\text{calc}}=\pi R_{x,\text{calc}}R_{y,\text{calc}}$, considering three different sizes of the lumen, defined by the angle $\Delta \theta_{\text{healthy}}$.

**FIGURE 8:**
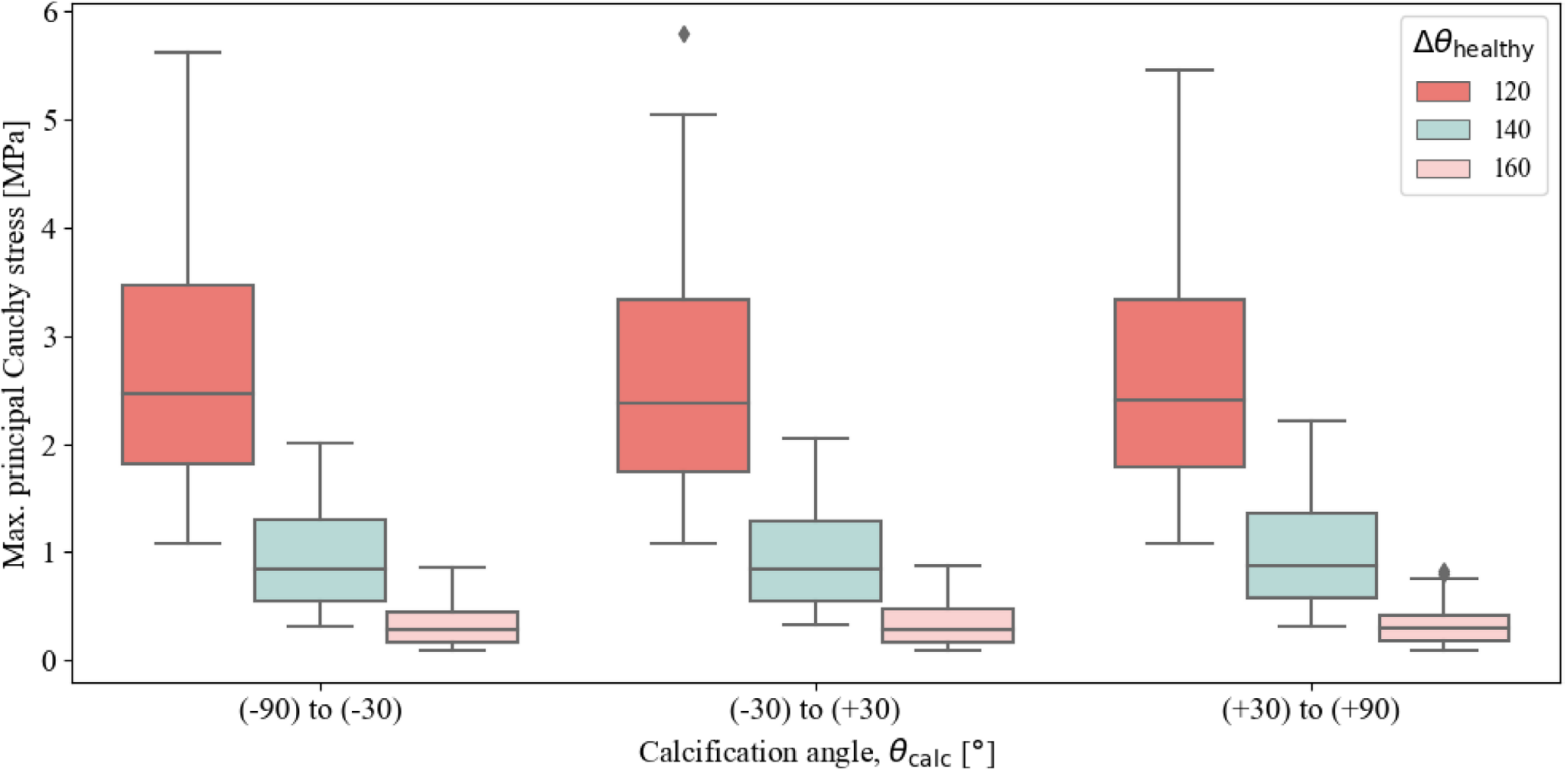
First principal Cauchy stress (absolute maximum) from all simulations, grouped by calcification angle $\theta_{\mathrm{calc}}$, considering three different sizes of the lumen, defined by the angle $\Delta \theta_{\text{healthy}}$.

Finally, we present the results on the influence of calcification size and location, modeled as a single ellipse with an area of $A_{\text{calc}}=\pi R_{x,\text{calc}}R_{y,\text{calc}}$. Figure 7 suggests that larger calcifications lead to slightly lower stress values than smaller ones, although this difference remains minimal. The stress reduction is more pronounced at $\Delta \theta_{\text{healthy}}=120{}^{\circ}$ and tends to disappear at large angles. The influence of the angular position of the calcification $\theta_{\text{calc}}$, illustrated in Figure 8, appears to be even less pronounced, suggesting that calcification had only a minimal effect on the maximum stress values in the artery. Again, the results are most strongly influenced by a change in the angle $\Delta \theta_{\text{healthy}}$.

## 4 Discussion

Understanding the mechanical behavior of atherosclerotic arteries during interventions such as balloon angioplasty is essential for improving treatment outcomes and minimizing complications, including plaque rupture. In this study, we developed a parameterized cross‐sectional model to simulate balloon inflation in atherosclerotic iliac arteries. Our focus was on assessing the sensitivity of the results to various geometrical parameters. Our results provide valuable insights into how factors such as the calcification size and location, the thickness of the fibrous cap, and the size of the lipid pool influence stress concentrations in the artery, which can serve as potential initiation sites for tissue damage and plaque rupture.

First, the influence of the fibrous cap thickness, represented by the parameter $T_{\text{fcap}}$, was investigated. Our analysis revealed that the maximum principal Cauchy stress is more likely to occur at the plaque shoulder. At the top of the fibrous cap, it occurs in less than ∼2% of simulations, and only when the fibrous cap is particularly thin. A comparison of these findings with pathological images of atherosclerotic arteries from the literature [13] showed that these two locations—the plaque shoulders and top of the fibrous cap—are also the primary sites of damage initiation in atherosclerotic plaques. Our results further suggest that in models with a thinner fibrous cap, the maximum principal Cauchy stress is more likely to occur at the top of the fibrous cap, increasing the likelihood of damage initiation in this region. Figure 5 demonstrates that with increasing lipid pool, the thickness of the fibrous cap has an increasingly greater influence on the stress distribution, typically indicating a more advanced disease state. Although $T_{\text{fcap}}$ influences the magnitude of the maximum principal Cauchy stress, its impact on the location of the maximum principal Cauchy stress is even more pronounced.

Second, we examined the influence of lipid pool size by varying the fibrous cap parameter $R_{\text{fcap}}$ and the angle spanned by the healthy portion of the artery $\Delta \theta_{\text{healthy}}$. The results indicate that plaques with larger lipid pools, determined by a combination of the two parameters, lead to higher stress levels. As the maximum principal stress can serve as a key indicator of potential damage in atherosclerotic arteries, our findings suggest that plaques with larger lipid pools are more prone to rupture or other forms of tissue damage. This observation is consistent with clinical studies that have shown that vulnerable plaques, which are more likely to rupture and lead to cardiovascular events, have large lipid pools [15, 21].

Third, we examined the influence of calcification size and location. As atherosclerosis progresses, both micro and macrocalcifications can develop in arteries, with the amount and size of these calcified regions varying depending on the artery and disease stage. In this study, we focused specifically on the inclusion of a single macrocalcification within the atherosclerotic plaque and examined variations in both its size and location. The results indicated that smaller calcifications had a moderate effect, slightly increasing the maximum principal Cauchy stress, especially in simulations with a reduced $\Delta \theta_{\text{healthy}}$. However, changing the position of the calcification had only a small influence on stress, suggesting a minimal influence on damage initiation. It is important to emphasize that our study focused exclusively on the effects of changes in the size and location of macrocalcification; we did not investigate the effects of balloon angioplasty with and without calcification. To further improve our parametric model, it would be useful to include microcalcifications, particularly within the fibrous cap, where they are commonly observed [10, 54]. Future studies should investigate the effects of the location, number, and size of microcalcifications by incorporating them into the parametric model to gain deeper insight into their potential role in damage initiation.

Finally, we would like to address further limitations of this work. The material model used to represent the behavior of the atherosclerotic artery contains structural parameters that characterize the angle and dispersion of collagen fibers in the tissue. To ensure that the material model accurately reflects the behavior of the atherosclerotic tissue, the structural parameters should ideally be obtained using second‐harmonic imaging microscopy [55, 56]. However, the experimental data used in this study consisted exclusively of uniaxial extension tests [48]. This implies that all parameters had to be fitted and fiber dispersion was not taken into account. The focus of this study was on iliac arteries, whose viscoelastic material behavior of the arterial tissue can significantly influence the stress response [57]. Future research would benefit from new experiments in atherosclerotic iliac arteries that investigate both material and structural parameters using second‐harmonic imaging microscopy and biaxial extension tests based on multi‐step relaxation protocols [58]. Furthermore, the balloon model could be refined by including the dynamics of balloon folding and unfolding, as described in [35], to more realistically represent the inflation process. Further improvements could also be achieved by refining the constitutive balloon model using a larger set of experimental data on the polymeric materials used.

Additionally, this study employed a parameterized cross‐sectional model as a simplification of a 3D model. Although 3D modeling of atherosclerotic plaques provides a more detailed representation of the complex geometry and mechanics, we chose a parameterized cross‐sectional model due to its significantly lower computational cost. The cross‐sectional model allowed us to conduct extensive parametric studies with a sample size of 3000 simulations, which would have been computationally difficult in 3D. Furthermore, a cross‐sectional model simplifies the handling of artery‐balloon contact mechanics and avoids the complexity of defining and solving 3D contact constraints. This approach enabled a high degree of efficiency that would have been unattainable with 3D modeling given the computational effort.

In our simulations, we investigated the influence of six geometrical parameters on the simulation outcome and selected the parameter limits based on physical considerations. Although we assumed that all parameter combinations were equally likely, we did not consider that certain combinations might be more likely than others. The predictive accuracy of the model could be substantially improved by incorporating patient‐specific atherosclerotic geometries from relevant clinical databases. Furthermore, our study was limited to a sample size of 3000 simulations; increasing this sample size in future work could lead to more robust and reliable results. Finally, it should be mentioned that we only performed a local sensitivity analysis, examining the influence of each parameter in isolation on the maximum principal Cauchy stress. A global sensitivity analysis that captures the interactions between parameters could provide further valuable insights.

## 5 Conclusions

In summary, the results underscore the crucial influence of various geometrical parameters on the structural integrity of atherosclerotic plaques. The simulations suggest that the fibrous cap parameter $R_{\text{fcap}}$ and the angle spanned by the healthy portion of the artery $\Delta \theta_{\text{healthy}}$, have the greatest influence on plaque vulnerability. Plaques with a larger lipid pool, reflected in lower values of $R_{\text{fcap}}$ and $\Delta \theta_{\text{healthy}}$, are more frequently exposed to elevated stresses, increasing the risk of damage and potential rupture. In contrast, the thickness of the fibrous cap $T_{\text{fcap}}$ and the size of the calcifications showed little influence. Variations in $T_{\text{fcap}}$ mainly affecting the location, rather than the magnitude, of the maximum principal stress. In particular, the location of the calcification angle $\theta_{\text{calc}}$ had no significant influence on stress concentration. These results highlight the importance of considering multiple geometric factors when assessing plaque stability and the need for further research on the combined effects of these parameters, particularly with regard to microcalcifications and their role in stress concentration and plaque rupture.

## Ethics Statement

The authors have nothing to report.

## Conflicts of Interest

The authors declare no conflicts of interest.

## Supporting information


**Data S1.** cnm70058‐sup‐0001‐Supinfo.

## Data Availability

The data that support the findings of this study are available from the corresponding author upon reasonable request.

## Endnotes

^1^
Like observed in Holzapfel and Ogden [47], the correct implementation of the fiber compression switch to ensure poly‐convexity in (6) is based on the fiber family invariant $I_{4}=U^{2}:\left(M⊗M\right)<1$. The modified condition is here presented for consistency with the implementation in the Abaqus/Standard FEA software [40].

## Appendix A

Figure A1 shows a collection of cross‐sectional geometries resulting from extreme parameter combinations. The first column refers to the considered geometrical region and the relevant geometrical parameters, whereas the second and third columns show the corresponding geometric outlines.
