# Supplementary material for: A Parameterized Cross‐Sectional Model for Simulating Balloon Angioplasty in Atherosclerotic Arteries
Source: Int J Numer Method Biomed Eng. 2025 Jul 18;41(7):e70058. doi: 10.1002/cnm.70058 (PMC12273774; doi:10.1002/cnm.70058)
Supplement: Supplementary file 1 — Data S1. cnm70058‐sup‐0001‐Supinfo. [file CNM-41-e70058-s001.pdf]

A Parameterized Cross-Sectional Model for Simulating Balloon Angioplasty in Atherosclerotic Arteries

S.M.B. Kwakman, M. Terzano, M. Rolf, G.A. Holzapfel

Supporting Information

1 Local material orientation

To correctly implement anisotropic material models, the basic vectors that define the respective material symmetry must be defined. Based on the theoretical framework presented in the manuscript, we illustrate here the computational procedure for the two-dimensional (2D) model of the atherosclerotic artery, implemented in the Abaqus Standard FEA software.

In the model proposed by Holzapfel et al. [1], the generalized structure tensor $\mathbf{H}$ of a single collagen fiber family is given by

$$\mathbf{H} = A\mathbf{I} + B\boldsymbol{M} \otimes \boldsymbol{M} + (1 - 3A - B)\boldsymbol{M}_{\mathrm{n}} \otimes \boldsymbol{M}_{\mathrm{n}} = H_{IJ}\boldsymbol{E}_I \otimes \boldsymbol{E}_J \,, \tag{1}$$

capturing the dispersion of the fibers in the reference configuration with respect to the mean fiber vector $\boldsymbol{M}$ and the out-of-plane unit vector $\boldsymbol{M}_{\mathrm{n}}$. The components of this tensor can be defined at the integration points of the finite element model with respect to a local reference basis $\{\boldsymbol{E}_I\}_{I=1,2,3}$ (shown in Figure 2(b) of the article).

Abaqus Standard FEA offers several options for defining local orientations, which are described in detail in the software documentation [2]. The approach proposed here applies to general 2D and three-dimensional (3D) geometries and is implemented entirely using Abaqus Python application programming interface (API). It is based on solving one (in 2D) or two (in 3D) Laplacian problems with user-defined boundary conditions, such that the local orientations of the material are provided by the normalized flux vectors [3]. For the currently implemented cross-sectional model, we solve a heat transfer problem radially in the artery, such that the unit vector in the radial direction is derived from the heat fluxes at the elements. The governing equations can be summarized as follows:

$$\nabla^2\Phi = 0 \quad \text{in} \quad \Omega_0 \,, \tag{2a}$$

$$\Phi = 0 \quad \text{on} \quad \Gamma_{\mathrm{ext}} \,, \tag{2b}$$

$$\Phi = \bar{\Phi} \quad \text{on} \quad \Gamma_{\mathrm{int}} \,, \tag{2c}$$

where the auxiliary scalar Φ represents a temperature and the symbol $\nabla^2(\bullet) = \partial^2{}_{x_1x_1}(\bullet) + \partial^2{}_{x_2x_2}(\bullet)$ denotes the

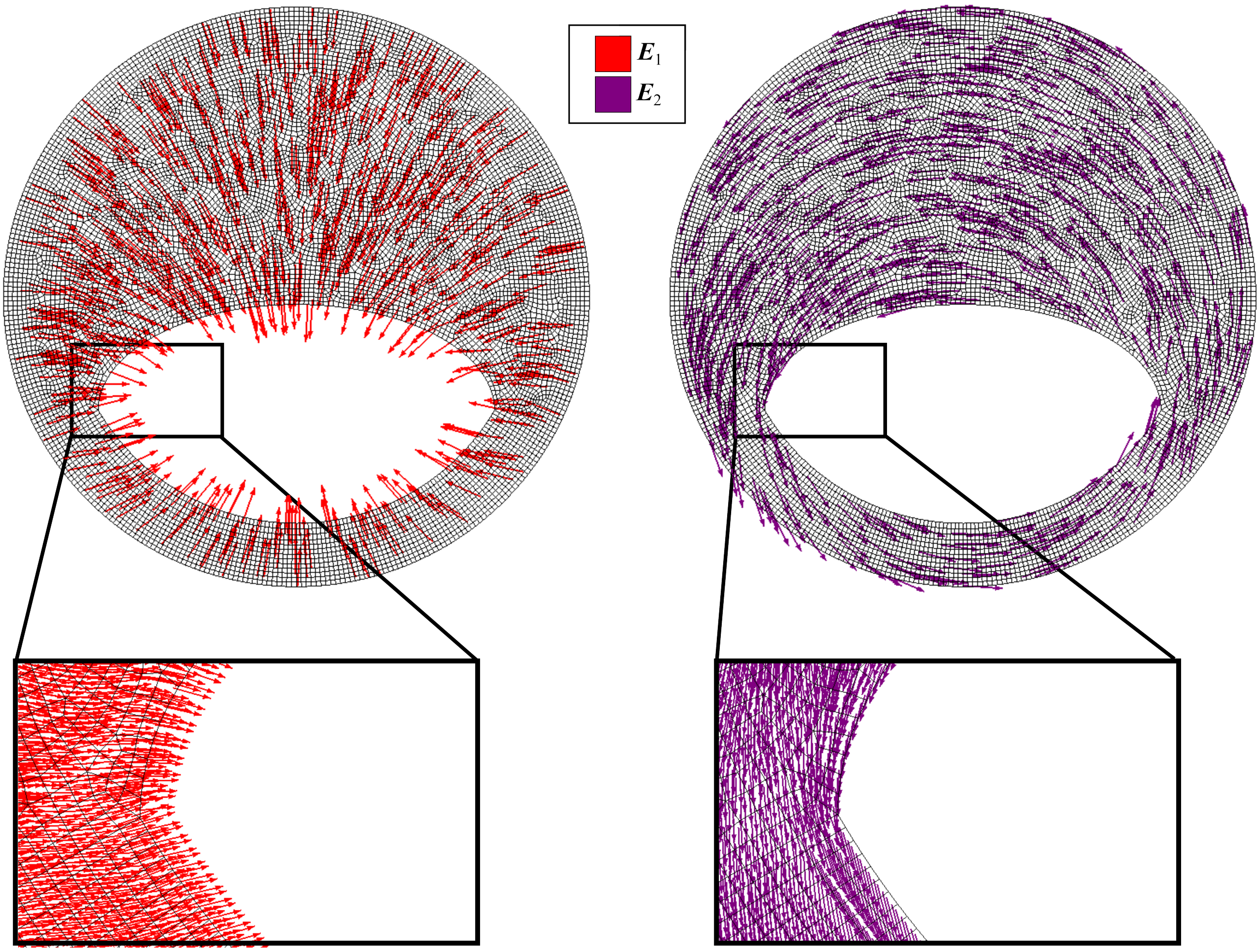

Figure 1: Left, radial local basis vector $\boldsymbol{E}_1$ and right, circumferential local basis vector $\boldsymbol{E}_2$. The geometrical parameters in these geometries are: $R_{\text{fcap}} = 2.8\,\text{mm}$, $T_{\text{fcap}} = 0.9\,\text{mm}$, $\theta_{\text{calc}} = 30°$, $R_{\text{x,calc}} = 1.5\,\text{mm}$, $R_{\text{y,calc}} = 0.5\,\text{mm}$, and $\Delta\theta_{\text{healthy}} = 120°$. The remaining fixed parameters are reported in the main article, see Table 2.

Laplace operator.

In (2), boundary conditions of the first type are imposed in terms of prescribed temperatures that define the values of the primary variable at the exterior surface Γ_{ext} to zero and at the interior surface Γ_{int} to a value $\bar{\Phi} > 0$. Note that the precise value of the temperature field variable is not relevant as long as a sufficiently large temperature gradient is generated in the domain.

The solution of the heat transfer analysis yields the heat flux vectors $\nabla\Phi$ at the centroid of each finite element, expressed in the global basis $\{\boldsymbol{G}_I\}_{I=1,2,3}$. The local reference basis vector $\boldsymbol{E}_1$ is derived from the radial heat flux; then, the circumferential direction $\boldsymbol{E}_2$ is calculated using basic vector algebra. By normalization, the basis vectors are

$$\boldsymbol{E}_1 = \frac{\nabla\Phi}{||\nabla\Phi||}, \qquad \boldsymbol{E}_2 = \boldsymbol{E}_1 \times \boldsymbol{E}_3, \qquad \boldsymbol{E}_3 = \boldsymbol{G}_3. \tag{3}$$

The entire process must be carried out before performing a mechanical analysis with the anisotropic material model. Since the basis vectors obtained depend only on the geometry, the heat transfer analysis should be repeated if the geometry or mesh is changed. Figure 1 shows the obtained radial and circumferential directions

for one of the artery geometries.

2 Mesh convergence

As mentioned in Section 2.3 of the article, the atherosclerotic artery and the angioplasty balloon are meshed using plane strain elements (CPE4H bilinear displacement pressure elements in the Abaqus FEA library). To identify the optimal seeding size for the mesh definition, a convergence analysis was performed. These preliminary analyses were performed on one of the parameterized geometries. The analysis was performed on a geometry with a thin fibrous cap and a small lipid pool using the following parameters: $R_{\text{fcap}} = 4.1$ mm, $T_{\text{fcap}} = 0.33$ mm, $\theta_{\text{calc}} = 30°$, $R_{\text{x,calc}} = 1.5$ mm, $R_{\text{y,calc}} = 0.5$ mm, and $\Delta\theta_{\text{healthy}} = 120°$.

The simulations were carried out with a reduction in seed size from 0.20 mm to 0.10 mm, with the number of elements varying between 3 914 and 16 681, respectively. As a result, the principal Cauchy stress at the top of the fibrous cap was taken. The results of this mesh convergence are shown in Fig. 2, where the relative difference is plotted against the seed size. Starting with a seed size of 0.13 mm, the result appears to be unchanged, so this value was chosen as the seed size for all subsequent simulations. The results of this mesh convergence study indicate that the relative error is below 5% for all tested seed sizes and below 0.5% for seed sizes 0.13 mm and smaller. This confirms that the principal Cauchy stress stabilizes at seed sizes ≤ 0.13 mm,

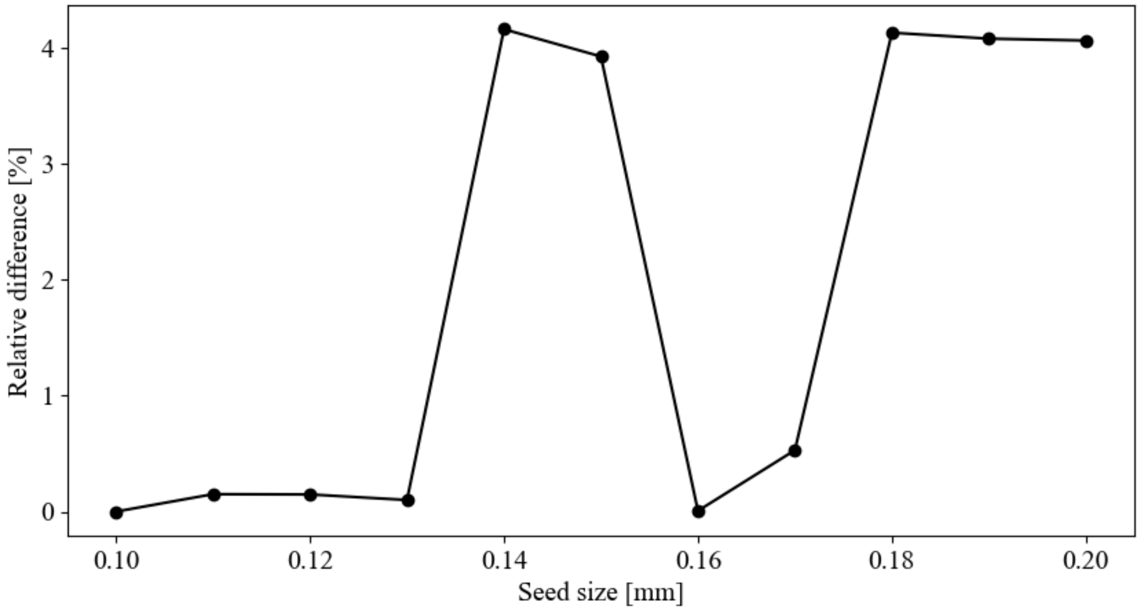

Figure 2: Mesh convergence of the atherosclerotic artery model. The relative difference in the principal Cauchy stress at the top of the fibrous cap is plotted against the seed size, with the solution at 0.1 mm serving as the reference solution.

References

[1] Gerhard A. Holzapfel et al. “Modelling non-symmetric collagen fibre dispersion in arterial walls”. In: *Journal of the Royal Society Interface* 12.106 (2015), p. 20150188. DOI: 10.1098/rsif.2015.0188.

[2] Dassault Systèmes SIMULIA. *Abaqus 2018, Software Documentation*. 2018.

[3] Richard Schussnig et al. “Semi-implicit fluid–structure interaction in biomedical applications”. In: *Computer Methods in Applied Mechanics and Engineering* 400 (2022), p. 115489. DOI: 10.1016/j.cma.2022.115489.
